# Measles and rubella seroprevalence in a population of young adult blood donors, France 2013

**DOI:** 10.1017/S0950268819000050

**Published:** 2019-03-01

**Authors:** D. Antona, P. Morel, C. Jacquot, L. Fonteneau, J. Dina, C. Vauloup-Fellous, L. Gimeno, A. Degeorges, P. Gallian, D. Lévy-Bruhl

**Affiliations:** 1 Direction des maladies infectieuses, Santé publique France, Saint-Maurice, France; 2 Etablissement Français du Sang, Paris, France; 3 Normandie Univ, UNICAEN, GRAM EA2556, 14032 France National Reference Center for Measles, Mumps and Rubella, CHU Caen, Virology Department, Caen 14000, France; 4 Department of Virology, AP-HP, Hôpital Paul Brousse, WHO Rubella NRL, Paris-Sud Univ, INSERM U1193, Villejuif 94804, France; 5 Etablissement Français du Sang Alpes Méditerranée, Marseille, France

**Keywords:** France, measles (rubeola), rubella, seroprevalence

## Abstract

As part of the evaluation of the French plan for the elimination of measles and rubella, we conducted a seroprevalence survey in 2013, aimed at updating seroprevalence data for people 18–32 years old. A secondary objective was to estimate measles incidence in this population during the 2009–2011 outbreak, and thus estimate the exhaustiveness of measles mandatory reporting. We used a cross-sectional survey design, targeting blood donors 18–32 years old, living in France since 2009, who came to give blood in a blood collecting site. We included 4647 people in metropolitan France, 806 people in Réunion Island and 496 in the French Caribbean. A further 3942 individuals were interviewed in the south-east region of metropolitan France to estimate the exhaustiveness of measles mandatory reporting. One of the main findings of this survey is that the proportion of people 18–32 years old susceptible to both measles and rubella infections remained high in France in 2013, 9.2% and 5.4%, respectively, in metropolitan France, even after the promotion campaigns about vaccination catch-up during and following the major measles epidemic in 2009–2011. Applying our results to French census data would suggest that around 1 million people aged 18–32 years old are currently susceptible to measles in France, despite this age group being one of the vaccination targets of the national measles elimination plan. Another important finding is that only an estimated 45% of the true number of cases in this age group was actually notified, despite notification being mandatory.

## Context

Measles and rubella infections are two major public health threats at the global level, and are targeted by the World Health Organization (WHO) for elimination. In the European region, the elimination target was initially planned for 2010 then postponed to 2015 [1, 2], without success. In 2014, during its 64th session, the WHO Regional Committee for Europe endorsed the European Vaccine Action Plan 2015–2020, aiming at measles and rubella elimination certified by the Regional Verification Committee for all European countries by 2018 [3].

In France, a national elimination plan was implemented in 2005 in order to meet the WHO European region's initial targets [4]. Despite the use of a combined measles-mumps-rubella vaccine (MMR) in the infant immunisation schedule for more than 30 years, France, as many other European countries, experienced a major measles outbreak in 2009–2011, clearly showing that the elimination goal was not yet reached. During this outbreak, half of the cases were observed in young adults aged 20–29 years [5]. In this age group, a high proportion (27%) occurred in people who received only one MMR injection. Although adolescent vaccine coverage (VC) reached 95% and 84%, for MMR1 and MMR2, respectively [6], data were lacking for VC in the population of young adults over 18 years of age. Furthermore, the immunity level in young adults remained too low, according to the results of a national seroprevalence survey conducted in 2009–2010, with 9% of 20–29 years old being susceptible to measles [7]. Consequently, in 2011, the French health authorities decided to recommend two doses of MMR for anyone born from 1980 onwards [8].

Among the key indicators selected by the WHO to assess measles and rubella elimination, an age-specific susceptibility target were set: in individuals aged 10 years and over, <5% should remain susceptible to measles and rubella infections [9]. As part of the evaluation of the French elimination plan, we conducted a seroprevalence survey targeted at young adults. This survey aimed at updating the seroprevalence data in people 18–32 years old, following the large 2011 measles outbreak and the subsequent measures implemented to increase VC for measles and rubella [5]. The second objective was to estimate measles incidence in this population during the 2009–2011 outbreak, and thus estimate the exhaustiveness of measles mandatory reporting.

## Methods

### Survey design and data collection

We designed a cross-sectional seroprevalence survey targeting blood donors 18–32 years old, living in France since 2009 and coming to give blood in a blood-collecting site during autumn 2013.

The French National Blood Service, sole provider of blood transfusion services in France, is organised into 17 regional centres: 14 in metropolitan France and three in French overseas departments (Islands of Martinique and Guadeloupe in the French Caribbean, and La Réunion in the Indian Ocean). All blood donations are made on a voluntary basis without financial incentive. Blood donors are required to complete a standardised medical questionnaire and are interviewed before giving blood, in order to ensure they meet donating criteria. During our study, donors were given documents containing information about the survey. Those who provided informed consent to participate were interviewed.

We used multistage, stratified sampling. In the first stage, the whole French territory was stratified into seven regions using phone number area codes (five area codes in metropolitan France and two for the overseas departments) and then within each region, according to type of blood collecting site (mobile or stationary). Sites were selected with unequal probabilities according to their expected activity, defined as the number of expected blood donors based on past activity. Sampling fractions were chosen to over-sample mobile sites in order to ascertain the inclusion of sparsely populated and remote areas, as well as sites like high schools, universities and companies where blood collections are regularly organised.

In the second stage, participants were included consecutively as they arrived at the blood collecting site. In each site, either four (less active sites) or eight donors (more active sites) were included with an equal number for the two age groups 18–25 and 26–32 years old, irrespective of gender. A supplementary blood sample was taken from people who agreed to participate in the survey. A face-to-face questionnaire was administered, documenting socio-demographic data (age, sex, professional activity, education level) as well as past and recent (since 2009) history of measles infection and vaccination. For each participant, both the blood sample and questionnaire were anonymised using unique survey codes, different from those used by the national blood bank.

Exclusion criteria were as follows: aged >32 years, exclusion from blood donation, not providing written consent to participate in the survey.

Based on a previous estimate of 9% for 20–29 years old susceptible to measles or rubella [8] and a desired precision level of 2%, the sample size needed was estimated at 5000 individuals for metropolitan France (1000 per region), 600–700 for Réunion, and 500–700 for the French Caribbean (Martinique and Guadeloupe).

The questionnaire was administrated during a face-to-face interview, and included the following socio-demographic variables: age, sex, place of residence, socio-professional category, education level. Data on history of past measles infection and vaccination status were collected on recall (for further details, the survey protocol – in French– can be sent by the authors upon request).

In order to meet the second objective of the survey, i.e. estimation of actual measles incidence and case under-reporting, the south-east region of metropolitan France was over-sampled, as it was by far the region with the highest incidence in 2011. Therefore, we interviewed (without taking blood samples) additional blood donors using the same questionnaire. To evaluate measles incidence, assuming under-reporting at 25%, the sample size needed was estimated at 5000 individuals.

### Laboratory methods

The regional biobanks of the French National Blood Service performed the pre-analytical processing, stored the samples and then sent the sera for analysis to the Alpes Méditérranée French Blood Service Laboratory in Marseilles as a one-time shipment after the last inclusion. Following the guidelines of the National Reference Laboratories (NRL) for measles (Caen University hospital) and rubella (Villejuif, Paul Brousse University Hospital), the sera were tested using an enzyme-linked immune assay (Anti-Measles Virus/IgG and Anti-Rubella-Virus/IgG Enzygnost microplate tests, Siemens Healthcare Diagnostics Products GmbH, Marburg, Germany) and single lot reagents.

Testing was performed according to the manufacturer's instructions. Prior to the beginning of the survey, the techniques were tested on panels validated by the two NRLs. The index value used to consider a sample negative was an IgG antibody concentration <150 mIU/ml for measles and <4 IU/ml for rubella. In order to compare titres, we used the IgG geometric means, and the logarithm of the results to plot the distribution of antibodies titres.

We compared our results with those of the seroprevalence survey conducted in 2010 in the general population [8]. For the latter, we took into account the cohorts of people who would turn 18–32 years old at the time of the 2013 survey. To compare rubella results, as the Elisa test used in the 2010 study was different (Access Rubella IgG, Beckman Coulter Inc., Brea, California, USA), the Siemens IgG Enzygnost cut-off point was chosen to evaluate whether a 2010 specimen was negative for rubella.

### Statistical analysis

A sampling weight was associated with each individual, calculated as the inverse of probability of inclusion. All statistical analyses were performed using Stata version 12.0 (StataCorp, College Station, Texas, USA) taking into account the sampling design. We improved the estimates by post-stratification on age group, sex, professional category and region of residence, according to the 2010 French census data (National Institute of Statistics and Economic Studies, INSEE).

Seroprevalence estimates were compared using the Pearson *χ*^2^ test and we calculated prevalence ratios (PR). We used a Poisson regression model to identify independent predictors of measles or rubella seronegativity while adjusting for multiple covariates and estimated adjusted PR. Explanatory variables for which a *P*-value of ⩽0.20 was found in univariate analysis were kept in the final multivariable model. But variables of epidemiologic interest such as socio-professional category or region of residency were finally forced into the models despite not being significant. First-order interactions were examined for statistical significance, epidemiologic plausibility and the impact of their inclusion on the other model parameters. The *F* test was used to assess the statistical significance of variables and interactions in the models and the models’ goodness of fit.

Student's *t* test was used to compare the geometric mean titres (GMT) according to individual age groups and history of vaccination or measles infection. To estimate the under-reporting rate of mandatory notifications, we compared the incidence estimates obtained in the south-east region of metropolitan France in the survey, with the rates of measles cases notifications per 100 000 population between 2009 and September 2013 in the same area, among the population aged 18–32 years at the time of the survey. We used France 2010 census data (INSEE) to calculate notification rates.

### Ethical and financial aspects

The survey protocol was reviewed and approved by a national institutional and ethical review board, confirming that anonymity of confidential information had been preserved.

The field survey and the serological analysis were funded by Santé publique France, the French National Public Health agency, management of field interviews as well as all the logistical aspects were run by the staff of the French National Blood Service.

## Results

Survey inclusion and data collection started on 23 September 2013 and lasted until 25 October 2013 in metropolitan France and 29 November 2013 overseas.

In metropolitan France, questionnaires and blood samples were obtained from 4647 people (four blood samples could not be tested for measles). Twenty-two individuals were excluded from the survey (age>32). In the south-east region alone, 1010 both completed the questionnaire and provided a blood sample. A further 3942 only completed the questionnaire (4952 in total).

Overseas, 806 people were included in Réunion Island (37 excluded because of their age). In the French Caribbean, 496 were included (17 excluded because of their age): 404 participants in Martinique, but only 92 in Guadeloupe, as blood collection had to be interrupted due to staffing problems. Therefore, data from Guadeloupe could only be analysed together with those from Martinique, and consequently, results are presented jointly for these French Caribbean islands.

### Measles

#### Susceptibility to measles

The overall estimates of the proportion of people seronegative for measles IgG antibodies were 9.2 (95% CI 7.9–11) in metropolitan France, 8.7% (95% CI 6.7–11.2) in Réunion and 7.7% (95% CI 4.6–12.6) in the French Caribbean.

We present both the univariate and multivariate analysis for metropolitan France in Table 1.

**Table 1.** Susceptibility to measles infection, French metropolitan population of blood donors aged 18–32 years, 2013

Measles	Proportion of those susceptible			Univariate analysis			Multivariate analysis		
	People tested (*n*)	% Seronegative	95% CI	PR	95% CI	*P*	PR	95% CI	*P*
Total	**4643**	9.2	(7.9–11.0)						
Age groups	**4643**								
18–25 years old	2515	10.0	(8.3–12.1)	1			1		
26–32 years old	2138	8.6	(7.9–10.7)	0.85	(0.63–1.14)	0.32	0.90	(0.65–1.28)	0.59
Gender	**4643**								
Men	2036	9.5	(7.7–11.6)	1			1		
Women	2607	9.0	(7.3–11.1)	0.95	(0.71–1.28)	0.78	0. 98	(0.73–1.32)	0.89
History of vaccination or past measles infection	**4638**								
History of measles infection	415	5.0	(3.0- 8.2)	1			1		
No past infection but vaccinated	2535	8.5	(7.0–10.5)	1.73	(1.01–2.98)	**0.04**	1.66	(0.97–2.84)	0.06
No or unknown past infection, and unknown vaccination status	1614	11.0	(8.6–13.8)	2.21	(1.26–3.87)	**0.006**	2.14	(1.22–3.75)	**0.008**
No past infection and unvaccinated	79	17.6	(9.7–29.9)	3.55	(1.66–7.58)	**0.001**	3.41	(1.59–7.31)	**0.002**
Education level	**4643**								
Elementary/middle school	786	8.8	(6.0–13.7)	1			1		
High school	2208	9.2	(7.4–11.4)	1.05	(0.66–1.66)	0.86	1.01	(0.65–1.58)	0.94
⩾2 years of higher education	1649	9.1	(7.3–11.4)	1.04	(0.67–1.63)	0.87	1.22	(0.78–1.93)	0.38
Socio-professional category	**4575**								
Farmers, craftsmen, workers	401	9.6	(6.0–15.0)	1			1		
Employees	1479	7.1	(5.5–9.2)	0.74	(0.43–1.26)	0.28	0.65	(0.37–1.17)	0.15
Managers, intermediate occup.	1150	9.7	(7.9–11.9)	1.01	(0.61–1.68)	0.95	0.97	(0.59–1.61)	0.92
Students	1197	10.0	(7.6–13.1)	1.04	(0.61–1.78)	0.89	0.99	(0.49–1.98)	0.97
Inactive	348	11.1	(6.7–17.6)	1.15	(0.58–2.26)	0.65	0.92	(0.55–1.55)	0.76
Region of residence	**4643**								
Paris and suburbs	880	11.2	(8.2–15.0)	1			1		
North-west	893	7.6	(5.5–10.4)	0.68	(0.43–1.06)	**0.09**	0.73	(0.47–1.15)	0.18
North-east	948	10.9	(8.1–14.6)	0.98	(0.64–1.49)	0.91	1.04	(0.67–1.60)	0.87
South-east	1014	7.0	(5.1- 9.5)	0.63	(0.41–0.97)	**0.04**	0.65	(0.41–1.02)	0.06
South-west	908	9.6	(6.1–14.8)	0.86	(0.50–1.47)	0.58	0.91	(0.52–1.57)	0.72

PR, prevalence ratio.

**In bold**: significant results (*P* < 0.20 in univariate analysis and *P* < 0.05 in multivariate analysis).

In univariate analysis, estimates were not significantly different for age, gender, education level, professional status or residence. The proportion of those susceptible to measles infection was significantly lower in people with a history of measles infection (5%, 95% CI 3.0–8.6) than in vaccinated people with no infection history (8.5%, 95% CI 7.0–10.5), in people with both a history of infection and unknown vaccination status (11.0%, 95% CI 8.6–13.8), and in people with no history of infection or vaccination (17.6%, 95% CI 9.7–29.9) (*P* < 0,05). Only 14 people had recently had measles and all were seropositive. As no difference was observed in susceptibility between people vaccinated before 2009 and those vaccinated after 2009 (2418 *vs.* 313), these data were combined in the analysis. People living in the south-east of metropolitan France were the least susceptible to measles infection: 7% (95% CI 5.1–9.5) (*P* = 0.04).

All the variables of interest in univariate analysis were retained in multivariate analysis, despite the fact that some were not significant independent predictors of susceptibility to measles. In multivariate analysis, only two factors remained significantly associated with a negative measles test result: (i) having no or an unknown history of measles, together with unknown vaccination status (PR = 2.14, 95% CI 1.22–3.75, *P* = 0.008) and (ii) no past infection or vaccination (PR = 3.41, 95% CI 1.59–7.31, *P* = 0.002). We did not find any significant terms of interaction between variables.

We compared our results with those of the seroprevalence survey conducted in 2010, prior to the 2011 peak of the measles outbreak [8]. As shown in Table 2, susceptibility to measles infection remained stable over time, without any significant difference between the results of the two surveys, even in the south-east region where the highest measles incidence was observed in 2011.

**Table 2.** Susceptibility to measles infection: comparison between the results of the 2010 and 2013 seroprevalence surveys, French metropolitan population aged 18–32 years

Measles	2010 Séro-Inf survey (*n* = 2322) Cohorts aged 18–32 in 2013		2013 Blood donors survey (*n* = 4643)		
	% Seronegative	95% CI	% Seronegative	95% CI	*P*
Total	8.3	6.8–10.1	9.2	7.9–10.7	NS
Age groups					
18–25 years old	7.3	5.5–9.7	10.0	8.3–12.1	NS
26–32 years old	9.4	6.9–12.6	8.5	7.9–10.7	NS
Gender					
Men	9.6	7.3–12.6	9.5	7.7–11.6	NS
Women	6.9	5.4–8.9	9.0	7.3–11.1	NS
Region of residence					
Paris and suburbs	8.1	4.2–14.9	11.2	8.2–15.0	NS
North-west	7.0	4.6–10.6	7.6	5.5–10.4	NS
North-east	8.9	7.6–11.9	10.9	8.1–14.6	NS
South-east	8.5	6.2–11.5	7.0	5.1–9.5	NS
South-west	9.3	6.8–18.7	9.6	6.1–14.8	NS

NS: non-significant.

#### Measles antibody titres

**Fig. 1.**
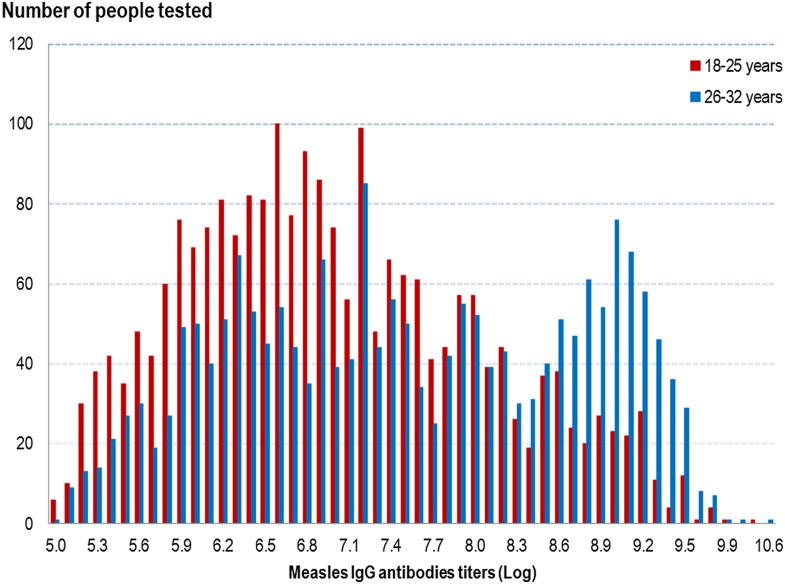
Distribution of measles IgG antibodies titres (Log) according to age group, French metropolitan population of blood donors aged 18–32 years, 2013.

Measles IgG titres were higher in people in the older age group, irrespective of a history of measles infection or vaccination, as shown in Figure 1. The overall GMT was 1083 mIU/ml (95% CI 1037–1131) in people aged 18–25 years and 1859 mIU/ml (95% CI 1762–1961) in people aged 26–32 years (*P* < 10^−5^) (cf. Table 3).

**Table 3.** Comparison of measles IgG GMT between age groups, according to past history of measles infection and vaccination, French metropolitan population of blood donors aged 18–32 years, 2013

Medical history	18–25 years				26−32 years				*P*
	*n*	GMT^a^	95% CI	*P*	*n*	GMT	95% CI	*P*	
Measles, irrespective of vaccination status	140	1744	1414–2149	Ref	275	4253	3752–4822	Ref	**<10^−5^**
No past history of measles and vaccinated	1387	1028	972–1087	0.003	1148	1575	1465–1694	<10^−4^	**<10^−5^**
No past history of measles and unknown vaccination status	940	1084	1009–1163	0.001	674	1737	1587–1901	<10^−4^	**<10^−5^**
No measles history and unvaccinated	48	1217	764–1939	NS	31	2016	1282–3168	0002	**NS**
Total	2515	1083	1037–1131		2128	1859	1762–1961		**<10^−5^**

NS: non-significant.

a GMT: measles IgG geometric mean titres, in mIU/ml

As shown in Table 3, in both age groups, the measles IgG GMT was significantly higher in people with a history of measles than in those with no past infection, irrespective of their vaccination history: 1744 mIU/ml (95% CI 1414–2149) in people aged 18–25 years and 4253 mIU/ml (95% CI 3752–4822) in people aged 26–32 years. Irrespective of age group, we found no GMT difference in people without a history of measles between those vaccinated and those who did not know their vaccine status. In people with no history of measles, we did not differentiate those vaccinated before from those vaccinated after 2009, as no difference in GMT was observed. Among the 14 people with a recent measles infection (infected since 2009), the IgG GMT was 7793 mIU/ml (95% CI 4650–13 059) in people aged 18–25 years and 9400 mIU/ml (95% CI 4877–18 118) in those aged 26–32 years (data not shown).

### Rubella

The overall estimates of the proportion of people testing seronegative for rubella IgG antibodies were significantly different between metropolitan France (5.4%, 95% CI 4.3–6.7) and overseas (1.3% (95% CI 0.6–2.5) and 2.0% (95% CI 0.9–4.2) in Réunion and the French Caribbean, respectively) (*P* < 0.02).

The detailed results for metropolitan France are shown in Table 4.

**Table 4.** Susceptibility to rubella infection, French metropolitan population of blood donors aged 18–32 years, 2013

Rubella	Proportion of those susceptible			Univariate analysis			Multivariate analysis		
	People tested (*n*)	% Seronégative	95% CI	PR	95% CI	*P*	PR	95% CI	*P*
Total	**4647**	5.4	(4.3–6.7)						
Age groups									
18–25 years old	2517	3.9	(2.7–5.6)	1			1		
26–32 years old	2130	6.7	(5.1–8.7)	1.72	(1.10–2.70)	**0.02**	1.6	(0.86–2.98)	0.14
Gender									
Men	2039	7.8	(6.1–10.0)	1			1		
Women	2608	3.1	(2.1–4.7)	0. 40	(0.25–0.64)	**0.0001**	0.44	(0.25–0.75)	**0.003**
Vaccination status	**4647**								
Vaccinated	2731	4.4	3.1–6.0	1			1		
Not vaccinated	158	8.8	4.9–15.1	2.01	(1.05–3.83)	**0.04**	1.97	(1.02–3.80)	**0.04**
Unknown	1757	6.6	4.9–8.9	1.52	(0.98–2.35)	**0.06**	1.33	(0.881–2.02)	0.17
Education level	**4647**								
Elementary/middle school	786	8.6	(4.2–12.5)	1			1		
High school	2211	4.1	(3.1–5.5)	0.47	(0.27–0.84)	**0.01**	0.57	(0.34–0.98)	**0.04**
⩾2 years higher education	1650	5.3	(3.9–7.3)	0.62	(0.34–1.12)	**0.11**	0.72	(0.41–1.27)	0.26
Socio-professional category	**4579**								
Farmers, craftsmen, workers	402	7.8	(4.1–14.2)	1			1		
Employees	1479	5.4	(4.1–7.0)	0.70	(0.35–1.38)	0.35	1.06	(0.52–2.15)	0.87
Managers, intermediate occup.	1153	5.5	(4.0–7.5)	0.71	(0.35–1.42)	0.30	0.95	(0.43–2.07)	0.88
Students	1197	3.9	(2.5–6.3)	0.51	(0.24–1.08)	**0.08**	0.95	(0.38–2.37)	0.91
Inactive	348	5.5	(2.6–11.3)	0.71	(0.27–1.85)	0.44	1.07	(0.37–3.06)	0.90
Region of residency	**4647**								
Paris and suburbs	880	6.2	(3.7–10.2)	1			1		
North-west	893	4.9	(2.7–8.6)	0.79	(0.37–1.71)	0.55	0.74	(0.34–1.63)	0.46
North-east	948	5.8	(3.8–9.0)	0.99	(0.51–1.95)	0.99	0.87	(0.43–1.76)	0.71
South-east	1018	4.5	(3.1–6.5)	0.72	(0.38–1.36)	0.30	0.66	(0.33–1.31)	0.24
South-west	908	5.5	(3.8–7.9)	0.88	(0.46–1.67)	0.70	0.82	(0.43–1.60)	0.57

PR, prevalence ratio.

**In bold**: significant results (*P* < 0.20 in univariate analysis and *P* < 0.05 in multivariate analysis).

In univariate analysis, the proportion of susceptible people was significantly lower in those vaccinated: 4.4% (95% CI 3.1–6.0) *vs.* 8.8% (95% CI 4.9–15.1) in unvaccinated people (*P* = 0.04). Susceptibility to rubella was significantly different when looking at the following variables: age (3.9% (95% CI 2.7–5.6) among 18–25 years old *vs.* 6.7% (95% CI 5.1–8.7) in people over 25) (*P* = 0.02), gender (3.1% (95% CI 2.4–4.7) in women *vs.* 7.8% (95% CI 6.1–10.0) in men) (*P* = 0.0001) and education level (8.6% among people with the lowest education level *vs.* 4.1% and 5.3%, respectively, for high school and university graduates) (*P* = 0.03). There was no significant difference in susceptibility between the different regions of residence.

In multivariate analysis, we did not find any significant terms of interaction between variables. The following factors were associated with lower susceptibility to rubella infection: female gender, vaccination against rubella and high school education (Table 4).

Just as for measles, we compared our results with those of the seroprevalence survey conducted in 2009–2010. As shown in Table 5, susceptibility remained stable over time and the only significant difference between the results of the two surveys was a decrease in the proportion of seronegative people in the south-east region.

**Table 5.** Susceptibility to rubella infection: comparison between the results of the 2010 and 2013 seroprevalence surveys, French metropolitan population aged 18–32 years

Rubella	2010 Séro-inf survey (*n* = 2322) Cohorts aged 18–32 in 2013		2013 Blood donors survey (*n* = 4647)		*P*
	% Seronegative	95% CI	% Seronegative	95% CI	
Total	4.3	3.4–5.5	5.4	4.3–6.7	NS
Age groups					
18–25 years old	2.5	1.6–3.9	3.9	2.7–5.6	NS
26–32 years old	6.5	4.6–8.9	6.7	5.1–8.7	NS
Gender					
Men	5.7	3.9–8.2	7.8	6.1–10.0	NS
Women	2.9	1.9–4.4	3.1	2.1–4.7	NS
Region of residence					
Paris and suburbs	4.3	2.1–8.5	6.2	3.7–10.2	NS
North-west	2.2	1.0–5.0	4.9	2.7–8.6	NS
North-east	3.0	2.0–4.7	5.8	3.8–9.0	NS
South-east	8.2	5.9–11.3	4.5	3.1–6.5	<0.05
South-west	3.0	1.2–7.3	5.5	3.8–7.9	NS

NS: non-significant.

#### Rubella antibody titres

**Fig. 2.**
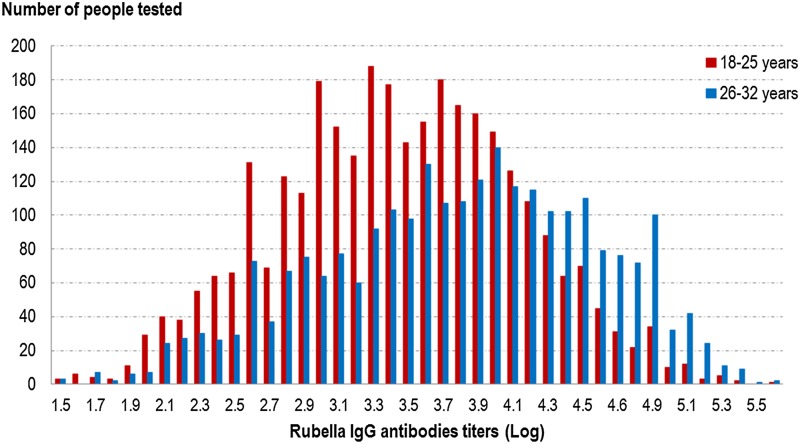
Distribution of rubella IgG antibodies titres (Log) according to age group, French metropolitan population of blood donors aged 18–32 years, 2013.

Globally, the GMT was significantly higher (*P* < 10^−5^) in the 26–32 years old group than the younger group (46.6 UI/ml (95% CI 45.0–48.2) *vs.* 31.0 UI/ml (95% CI 30.2–31.9)) (cf. Fig. 2 and Table 6). In the younger age group, the titres were not significantly different between vaccinated people, unvaccinated people and those with an unknown vaccination status. In the older group, unvaccinated people had significantly higher GMT (*P* < 0.05) than the other groups (cf. Table 6).

**Table 6.** Comparison of rubella IgG GMT between age groups, according to past history of rubella vaccination, French metropolitan population of blood donors aged 18–32 years, 2013

Rubella vaccination history	18–25 years old				26–32 years old				*P*
	*n*	GMT	95% CI	*P*	*n*	GMT	95% CI	*P*	
Vaccinated	1458	31.5	30.4–32.6	Ref	1273	45.6	43.7–47.6	Ref	<10^−5^
Unknown vaccination status	989	30.3	28.9–31.7	NS	769	46.5	43.9–49.3	NS	
Unvaccinated	70	33.5	27.4–41.1	NS	88	65.2	54.6–77.8	0.02	
Total	2517	31.0	30.2–31.9		2130	46.6	45.0–48.2		

As observed for measles, we did not find any difference in antibody titres when comparing people vaccinated before 2009 with those vaccinated after 2009.

### Estimation of measles incidence and completeness of reporting in the south-east of France

A total of 4952 people were interviewed in the south-east region during the survey: 3177 aged 18–25 years (64%) and 1775 aged 26–32 years. Of the 418 who indicated a history of measles infection, 29 cases occurred in or after 2009. Three of the latter declared that they did not visit a physician while they were sick.

After weighting and adjusting, the 2009–2013 cumulative measles incidence rate in the south-east region in people aged 18–32 years in 2013, was estimated at 307 cases per 100 000 inhabitants (95% CI 183–517) in people diagnosed by a physician. Most of them were people 18–25 years old, with a cumulative rate of 491 cases per 100 000 people *vs.* 143 cases per 100 000 people aged 26–32 years (cf. Table 7).

**Table 7.** Estimation of completeness of measles cases reporting

Age groups	South-east population (18–32 years)	Mandatory reporting		2013 Seroprevalence survey		Completeness of reporting	
		Measles notifications (*n*)	Notification rate (per 100 000)	Cumulative incidence rate (per 100 000)	95% CI	%	95% CI
18–25 years	13 06 178	2913	223	491^a^	264–910	45	25–84
26–32 years	14 35 664	1818	127	143^a^	55–374	89	34–>100
Total	27 41 842	4731	173	307^a^	183–517	56	33–94
				384^b^	214–688	45	25–77

a Estimate of the cumulative incidence rate in people diagnosed by a physician.

b Estimate of the total cumulative incidence rate, including patients who did not consult a physician.

A total of 4731 cases were reported to the Local Health Authority through the mandatory notification system between January 2009 and September 2013 in the 18–32 years old population living in the south-east of France at the time of the survey (i.e. during autumn 2013). Most of the cases (2913, 62%) occurred among people 18–25 years old, with a cumulative rate of 223 reported cases per 100 000 people *vs.* 127 reported cases per 100 000 people aged 26–32 years (cf. Table 7), corresponding to a cumulative rate of 173 notified cases per 100 000 inhabitants

The completeness of measles mandatory reporting was estimated at 56% (173/307) (95% CI 33–94). When taking into account cases who did not visit a physician, we estimated that only 45% (95% CI 25–77) of the measles cases that occurred in the community had actually been included in the official health statistics.

## Discussion

Our study was based on volunteer blood donors, which may limit the interpretation of epidemiological data, as in any other blood donor study. Due to donation rules, people <18 years of age were not included. Nor were those who had a history of a virus-like illness in the 28 days before donation. Furthermore, in terms of vaccination and prevention behaviours, it is not known how blood donors compare with the general population. Another limitation was the absence of documentation to rely on, both for vaccination status and history of measles. On the contrary, our study design allowed us to enrol a high number of individuals during a short period of time and to compare results from similar populations in different locations.

We did not find any significant difference in the overall measles seroprevalence estimates between metropolitan France and French overseas territories. More specifically, the proportion of people susceptible to infection ranged from 7.7% in the French Caribbean to 9.2% in metropolitan France, which does not meet the target of <5% set by the WHO European region for measles elimination for people aged 10 years and older [9]. In metropolitan France, we did not observe any significant difference in the prevalence estimates when looking at age, gender, education level, professional status and residence. In the univariate analysis, the proportion of those susceptible to infection was significantly lower in people living in the south-east of France (where the incidence of measles cases was the highest in 2011). However, this difference was no longer significant in multivariate analysis. Unsurprisingly, in our multivariate model, only two factors remained significantly associated with a negative result to measles testing: (i) unknown or no history of measles together with unknown vaccination status, and (ii) no past infection or vaccination.

Comparing our results with those of the seroprevalence survey conducted in 2009–2010 (i.e. prior to the 2011 measles outbreak peak), susceptibility to measles infection remained stable over time, no significant difference being observed between the two surveys. This was true even in the south-east region, which was the most affected by the epidemic. This would suggest that the number of young adults who became immune after contracting the disease during the outbreak, or, more importantly, who should have been targeted by the reinforced control measures, especially the two MMR dose catch-up, was not large enough to actually impact the immunity profile in this population. Only unrealistically large sample sizes would have been able to identify a possible slight increase in seroprevalence between the two surveys.

Measles IgG antibodies titres were significantly higher in older people, the overall GMT in people aged 26–32 years being twice that in people aged 18–25 years (cf. Table 3). In each age group, the measles IgG GMT was significantly higher in people with a history of measles. These results are not surprising as older people were more likely to have been infected during childhood, and as vaccine-induced antibody titres are lower than those achieved after natural infection [10, 11, 12]. In people with no history of measles, there was no GMT difference between those vaccinated (recently or not) and those who did not know their vaccine status, irrespective of age group. This may be because most people in the unknown vaccination status group were in fact vaccinated. As participants were unaware of the study before coming to the blood transfusion centre, they were not able to provide any documentation ascertaining their immunisation status or any history of measles. Consequently, no analysis of GMT levels according to time since vaccination or infection was possible.

The proportion of people susceptible to rubella infection was significantly different between metropolitan France and overseas territories (5.4% *vs.* 1.3% in Réunion and 2.0% in the French Caribbean (*P* < 0.02)). In metropolitan France, no significant difference was observed according to the region of residence. We had no information on history of rubella infection, but the proportion of seronegative individuals was significantly lower (by 50%) in those vaccinated than in those unvaccinated. In multivariate analysis, the factors associated with lower susceptibility to rubella infection were: female gender, MMR vaccination and high school education level. The difference in susceptibility we observed between men and women is not surprising, as in France, rubella vaccination was originally targeted only at girls to prevent congenital rubella syndrome, before being recommended to both boys and girls.

As observed with measles, comparison with the previous seroprevalence survey showed that the proportion of seronegative people remained stable over time. The only difference between the results of the 2009–2010 and 2013 sero-surveys was a significant decrease in this proportion in the south-east region.

We found that rubella IgG antibodies titres were significantly higher in older people. In this age group, the highest GMT was found in unvaccinated people. These results suggest a higher proportion of individuals with a history of past rubella infection as age increases. As for measles, irrespective of age, there was no difference in GMT between people vaccinated and those who were unaware of their vaccination status, suggesting a high proportion of vaccinated people in the latter group, especially as rubella circulation has been very low for more than a decade.

We estimated the 2009–2013 measles cumulative incidence rate in the south-east region in people aged 18–32 years in 2013, at 307 cases per 100 000 inhabitants. This result would mean that only 56% of the cases seen by the physicians had actually been reported to the health authorities. Taking into consideration the cases who did not visit a physician, we consequently estimated that only 45% of the total true number of cases that occurred in this population had been actually notified, despite notification being mandatory. In other words, although 4731 cases were notified to the health authorities in the 18–32 years old population in the south-east of France between 2009 and 2013, the actual figure was probably over 10 000 measles cases. At the same time and in the same age group, a total of 7605 cases were notified in metropolitan France. Under the hypothesis of an equivalent case reporting rate over the entire country for this age group, the total number of cases that occurred in the country in people aged 18–32 years during this period of time can be estimated at 16 731 cases (95% CI 9887–30 420). Such a finding confirms that the proportion of individuals that became infected over the course of the outbreak in 18–32 years old was much too low (25 per 10 000) to translate into a significant increase in seroprevalence that could be demonstrated through a study of reasonable size. If we assume that this 45% under-notification rate also applies to the other age groups, the 22 725 notified measles cases would translate into 50 500 actual cases.

## Conclusion

One of the main findings of this survey is that the proportions of people aged 18–32 years still susceptible to both measles and rubella infections remained high in France in 2013, even after the promotion of vaccination catch-up following the major measles epidemic in 2009–2011. Applying our results to the French census data would suggest that around 1 million people aged 18–32 years are currently susceptible to measles in France, despite this age group being one of the vaccination targets of the national measles elimination plan.

In France, the main issue regarding measles and rubella elimination is insufficient VC, levelling off at approximately 90% for the first dose but remaining below 80% for the second dose in 2-year-old children, with insufficient catch-up in older cohorts. It is likely that on-off catch-up vaccination campaigns could help increase VC, but these are still considered inappropriate in France, mainly in the context of at least two previous vaccination campaigns run by the Ministry of Health which resulted in increasing public vaccine hesitancy and disbelief in health authorities: a vaccination campaign against hepatitis B targeting teenagers, conducted in schools in 1995–1997, and the mass vaccination campaign against H1N1 influenza conducted in the general population in autumn 2009. The French Ministry of Health has therefore favoured regular measles vaccination promotion campaigns through mass media and widespread distribution of promotional materials targeting both health professionals and the general public. MMR vaccine has been made the only childhood vaccine fully free. The latest measure taken by the French Ministry of Health to improve coverage was to pass a law extending mandatory vaccination from three to 11 vaccines for infants, including MMR, starting from January 2018 [13]. However, it will take years for such a measure to allow eliminating measles in France, unless VC rapidly increases in older birth cohorts. One of the main challenges in this new context is therefore to convince the health professionals and the general public that catching up older unvaccinated children and young adults is as important as infant vaccination, although being only recommended.

With more than 2700 measles cases notified between January and August 2018, 50% of them being over 15 years of age [14], the elimination of measles in France is not yet achieved. Nevertheless, the experience with measles and rubella elimination in countries such as the USA, Australia and Finland shows that endemic transmission can be interrupted in a region if governments are committed to maintaining immunisation coverage above the immunity threshold, to providing high-quality surveillance and to implementing prompt control measures around imported cases [15, 16, 17].

Will France be in a situation to meet the 2020 measles elimination target? Our results suggest the persistence of a reservoir of susceptible individuals in 2013 in young adults, but the levels of susceptibility in people below 18 years of age should now be close to the age-specific WHO elimination thresholds, as the most recent MMR VC estimates are above 95% at 6, 11 and 15 years [18]. An important feature is the level of clustering of the remaining susceptible individuals. Estimation of measles vaccination coverage at sub-district level through the newly available exhaustive national vaccines’ reimbursement database will help to identify pockets of unvaccinated people. A modelling exercise including VC geographical heterogeneity is currently being carried out to assess the risk of future measles resurgence. However, in a near future, MMR VC should definitively benefit from the recent law making compulsory the infants’ vaccinations.
